# A Narrative Review of the Putative Etiologic Role and Diagnostic Utility of the Cervicovaginal Microbiome in Human Papillomavirus‐Associated Cervical Carcinogenesis

**DOI:** 10.1002/jmv.70027

**Published:** 2024-11-08

**Authors:** Margaret Logel, Parker Tope, Mariam El‐Zein, Emmanuel Gonzalez, Eduardo L. Franco

**Affiliations:** ^1^ Division of Cancer Epidemiology McGill University Montreal Quebec Canada; ^2^ Department of Human Genetics Microbiome Unit, Canadian Centre for Computational Genomics (C3G), McGill University Montreal Quebec Canada; ^3^ Centre for Microbiome Research McGill University, Montreal Montreal Quebec Canada

**Keywords:** cervical cancer, cervicovaginal microbiome, human papillomavirus

## Abstract

The cervicovaginal microbiome (CVM) may contribute to human papillomavirus (HPV)‐associated cervical carcinogenesis. We summarized the literature on the CVM in cervical carcinogenesis by searching Medline, Web of Science, and Embase for articles that sequenced the CVM using metagenomics. Additionally, we identified studies assessing the diagnostic role of the CVM in cervical carcinogenesis by searching PubMed. We performed an environmental scan of Google and Google Scholar to review common CVM characterization techniques. Twenty‐eight records presented or summarized associations between the CVM and HPV acquisition, prevalence, persistence, clearance, and cervical lesions or cancer, while three studies identified bacterial taxa detecting high‐risk HPV prevalence or cervical lesions. The area under the curve ranged from 0.802 to 0.952. 16S ribosomal RNA gene sequencing and whole metagenome sequencing have sufficient resolution to study the CVM bacteriome. Bacterial communities may have important implications in cervical cancer; however, there is a need for methodological standardization for CVM characterization.

## 1 Introduction

Human papillomavirus (HPV), the most common sexually transmitted infection (STI) [1], is a necessary cause of invasive cervical cancer and its precancerous lesion, cervical intraepithelial neoplasia (CIN) [2]. Globally, cervical cancer is the fourth most common female cancer despite being preventable with HPV vaccination and cervical cancer screening [3]. As of 2020, there were approximately 604 000 new cases and 342 000 attributable deaths [3]. Vaccination coverage is suboptimal across Canada (uptake rates < 80% in most provinces) [4] and in the United States (US) (uptake rates < 60%) [5]. Moreover, since the primary target age for HPV vaccination is pre‐adolescence and adolescence, most adults remain unvaccinated and at high risk of acquiring an HPV infection. Vaccination and screening uptake are inferior in low‐ and middle‐income countries; less than 30% have implemented vaccination programs, and only 44% of women have been screened for cervical cancer [3]. Although vaccination and screening will continue to be the main prevention strategies for cervical cancer, there is a need for continued research to reduce the disease burden.

An area of growing research interest is the relationship between the communities that constitute the cervicovaginal microbiome (CVM) and HPV, as well as the CVM and cervical cancer. The microbiome consists of several micro‐organisms, including archaea, bacteria, fungi, plasmids, and viruses. In particular, the bacteriome (i.e., bacterial community) has been shown to vary over time based on genetic, hormonal, and environmental factors [6, 7]. High concordance rates between microbes identified in cervical and vaginal samples from the same subjects suggest that the microbial composition at these anatomical sites is generally comparable [8]. Recent evidence suggests that the vaginal microbiome of a healthy female is dominated by bacterial species of the *Lactobacillus* genus corresponding to low microbial diversity [9]. Bacterial vaginosis (BV), also known as vaginal dysbiosis, is characterized by a loss of *Lactobacillus* dominance and an increase in anaerobic bacteria contributing to high species diversity [10]. This state has been associated with the acquisition of STIs, including gonorrhea and Chlamydia [11].

Thirteen high‐risk HPV (hrHPV) types are considered carcinogenic to the uterine cervix [2, 12]. Particularly, HPV16 and HPV18 cause 70% of cervical cancers [13]. Although necessary, hrHPV infections are not a sufficient cause, indicating the involvement of additional co‐factors along the causal pathway. The majority of HPV infections are cleared or kept latent by the immune system; it is the persistence of hrHPV that can lead to CIN lesions (which can spontaneously regress) and cervical cancer [14, 15, 16, 17]. Little is known about the biological factors and mechanisms that influence the prevalence, acquisition, persistence, and clearance of hrHPV infections and the progression and regression of CIN lesions. Meta‐analyses have suggested that CVM communities may play a role in HPV acquisition, prevalence, persistence, and clearance as well as the development of CIN lesions or cervical cancer [18, 19, 20].

Numerous microbial characterization techniques and bioinformatic pipelines of microbial community data exist and have evolved in technical sophistication, which enhanced our understanding of the CVM. Microscopic evaluation is utilized to identify BV; some techniques include the Nugent score (a gram‐staining method) [21], Amsel criteria [22], and the Papanicolaou smear for detecting clue cells [23]. The above methods are morphological and thus lack resolution. Molecular testing approaches are necessary to study the numerous aspects of the CVM some of which include microbial diversity, isolate identification of microbial communities, and the assessment of beta‐diversity between samples, among others. In this regard, metagenomics permits studying the community structure, activity, and functional potential of micro‐organisms in a biological sample. Several different metagenomic techniques exist, including, but not limited to, gene marker analysis, shotgun metagenomics, transcriptomics, proteomics, and metabolomics, all of which have different detection abilities, strengths, and limitations for identifying microbial communities [24]. Gene marker analysis is a targeted sequencing method. Specifically, for CVM characterization, 16S ribosomal RNA (16S rRNA) gene sequencing is a common technique that targets bacterial taxa. Ravel and colleagues were the first to identify community state types (CST) of the CVM in reproductive‐aged women using 16S rRNA gene sequencing [9].

In light of the critical need for continued research to reduce cervical cancer burden and the variation in CVM characterization methods, the primary objective of this review was to summarize the role and diagnostic potential of the CVM in HPV‐associated cervical carcinogenesis, with a focus on studies that characterized the CVM using metagenomic techniques. Our secondary objectives were to summarize findings from relevant systematic reviews and meta‐analyses and review 16S rRNA gene sequencing and whole metagenome sequencing (WMS, considered the gold standard technique for microbial characterization) in terms of methodological characteristics and CVM identification ability.

## 2 Materials and Methods

The current narrative review consisted of two components: (1) a systematic search of the literature to summarize the CVM's role in HPV‐associated cervical carcinogenesis, and (2) an environmental scan of Google and Google Scholar to summarize metagenomic techniques for CVM characterization. For simplicity, and due to the high concordance in bacterial diversity between the cervix and vagina as sampling sites [8], we considered the cervical and vaginal microbiome interchangeably (referred to as the CVM) without distinguishing between the two. Moreover, as the majority of literature on the topic focuses on the CVM bacteriome, we only considered the CVM bacteriome rather than the complete microbiome.

First, we identified original research articles, systematic reviews, and meta‐analyses on the role of the CVM in HPV‐associated cervical carcinogenesis by searching Medline, Embase, and Web of Science from inception to July 27, 2022, using MeSH headings and keywords related to “HPV,” “cervical cancer,” and “CVM.” Records later published were monitored in PubMed for relevance until October 13, 2023. The search strategies, reviewed by a McGill University librarian, are presented in Table S1. Records were managed in EndNote version X9, and duplicates were removed automatically and manually. Titles and abstracts were screened for relevance in Rayyan, and the full texts of relevant articles were independently assessed for eligibility by two reviewers (M.L. and P.T.). Disagreements were reviewed and resolved by consensus. To be eligible, original research articles had to characterize the CVM using a metagenomic technique and include a relative or absolute measure of association between the CVM and one or more outcomes of interest (HPV acquisition, prevalence, persistence, clearance, and cytology interpretations or biopsy‐confirmed CIN and cervical cancer). Meta‐analyses and systematic reviews had to discuss the relationship between the CVM and one or more of the outcomes of interest. We applied a language restriction, including only English publications. For research articles, we extracted the overall findings, exposures, outcomes, effect estimates and 95% confidence intervals (CI), and details regarding the study design, population, methods, microbial characterization, and HPV genotyping techniques. For systematic reviews and meta‐analyses, we extracted the general findings relating to the outcomes of interest, number of included studies, microbial characterization techniques of the included studies, and limitations. For meta‐analyses, we additionally extracted the inclusion/exclusion criteria, exposures, outcomes, pooled effect estimates, and their corresponding heterogeneity statistics. Effect estimates were considered statistically significant when the 95% CI did not include the null value of the outcome measure. For articles that did not report a 95% CI, *p* < 0.05 was considered statistically significant.

To identify studies that assessed the diagnostic performance of CVM components in HPV‐associated cervical carcinogenesis, we performed a separate PubMed search (by title and abstract) from inception until June 23, 2023, using keywords relating to “area under the curve (AUC),” “receiver operating characteristic (ROC),” “HPV,” “cervical cancer,” and “CVM” (Table S1). Eligible records included those that characterized the CVM with metagenomic techniques, assessed the diagnostic performance of the CVM to detect an outcome of interest using an ROC curve analysis, and reported an AUC and 95% CI. Record screening and data extraction (study design, population, microbial characterization methods, microbial components, and AUC with corresponding 95% CI) were performed by one reviewer (M.L.).

For the second component of the narrative review, we browsed Google and Google Scholar to conduct an environmental scan of 16S rRNA gene sequencing (most common metagenomic technique for CVM characterization) and WMS to provide an overview of metagenomic approaches in microbial research.

## 3 Results

**Figure 1:**
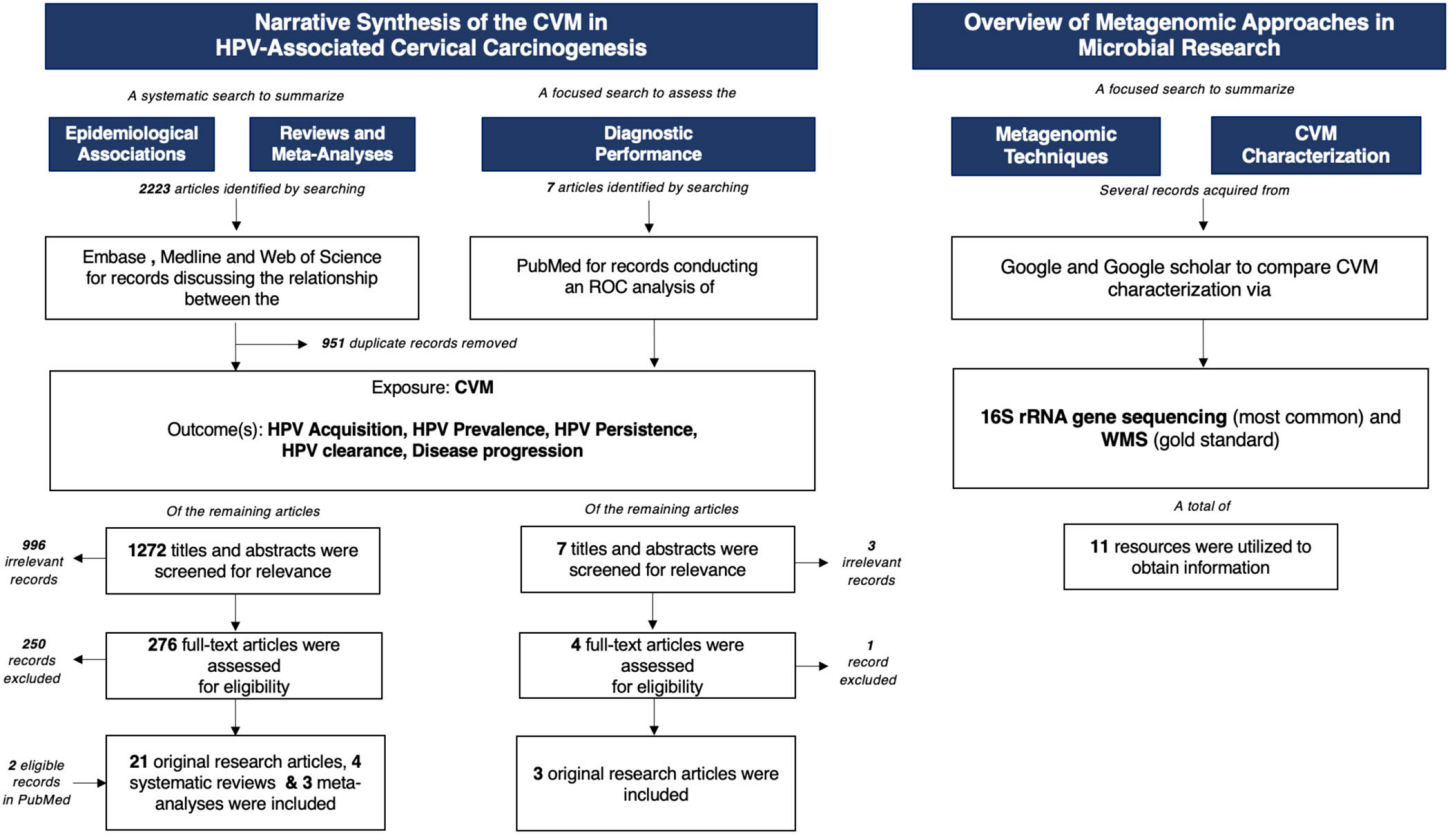
Mapping of the search strategy, methodology, and results. To assess the role of the CVM in HPV‐associated cervical carcinogenesis, 2223 records were identified in Embase, Medline and Web of Science, of which 951 duplicates were excluded. 1272 articles were screened by title and abstract for relevance and the full texts of 276 articles were assessed for eligibility. Two additional articles were identified in PubMed following the systematic search. A total of 21 observational studies, 4 systematic reviews, and 3 meta‐analyses were included. To assess the diagnostic performance of the CVM in HPV‐associated cervical carcinogenesis, 7 relevant articles were identified in PubMed, of which 3 were excluded. Of the 4 full texts assessed for eligibility, one was excluded as it only assessed the diagnostic performance of bacterial genes (not taxa). The corresponding search strategies for the aforementioned searches are detailed in Table S1. To identify common metagenomic techniques in microbial research, 11 resources were identified through a focused search of Google and Google Scholar to identify webpages and scholarly articles, respectively. Abbreviations: CVM, cervicovaginal microbiome; HPV, human papillomavirus; ROC, receiver operating characteristic; WMS, whole metagenome sequencing; 16S rRNA, 16S ribosomal RNA.

The overall methodology is shown in Figure 1. Following full‐text review of 276 articles, 26 were deemed eligible; two additional articles were identified in PubMed following the systematic search for a total of 28 included articles. Of the seven articles identified in PubMed on the diagnostic performance of the CVM in HPV‐associated cervical carcinogenesis, three were eligible following primary and secondary screening. Additionally, information on 16S rRNA gene sequencing and WMS was acquired from 11 webpages/scholarly articles identified in Google (webpages) or Google Scholar (scholarly articles).

### 3.1 Etiologic Role of the CVM in HPV‐Associated Cervical Carcinogenesis

Detailed information on the 21 observational studies presenting epidemiological associations is shown in Table S2 whereas Table 1 lists the main findings. All records were published between 2014 and 2023. Studies were conducted in populations from Brazil [36], Canada [27], China [29, 38], Costa Rica [28, 34], Italy [39], Korea [33, 37, 44], Mexico [40], Nigeria [25, 30, 41], Portugal [26], South Africa [35], and the US [31, 32, 42, 43, 45]. Sample size of included studies ranged from 12 to 807 women. Measures of association were reported between the CVM and HPV prevalence (*n* = 8), acquisition (*n* = 4), persistence (*n* = 4), and clearance (*n* = 4), and between the CVM and cytological interpretations or biopsy‐confirmed CIN/cervical cancer (*n* = 13). Most studies were cross‐sectional (*n* = 10) and longitudinal (*n* = 9) with two nested‐case–control studies. The majority (*n* = 18) utilized 16S rRNA gene sequencing for CVM characterization, differing by the hypervariable regions amplified (V1–V2, V1–V3, V3–V4, V3–V5, V3–V6, or V4) and sequencing platforms utilized (Genome Sequencer Titanium Roche‐454, Illumina HiSeq, Illumina MiSeq, NovaSeq). The remaining three studies characterized the CVM using either polymerase chain reaction (PCR) amplification, Allplex Bacterian Vaginosis Assay, or 16S ribosomal DNA (16S rDNA) gene sequencing. Specific study findings grouped by the outcomes of interest are detailed below.

**Table 1:** Observational studies on the association between the CVM and HPV‐associated cervical carcinogenesis.

References	Study design (number of visits)	Country	Outcome(s) assessed^a^	Main findings
[25]	Cross‐sectional	Nigeria	Cytology or biopsy results	‐No significant association found between any CST types and HSIL/ICC versus NILM and LSIL cytology in the entire study population or following stratification by HPV positivity
[26]	Cross‐sectional	Portugal	HPV prevalence; cytology or biopsy results	*‐Lactobacillus* species, *Gardnerella vaginalis*, and BV panel found to significantly decrease the risk of the presence of multiple hrHPVs, HPV16 or HPV18, and 9‐valent HPVs whereas *Mobiluncus* species significantly increased the risk of 9‐valent HPVs Following co‐variate adjustment, in three multivariable models: ‐*Lactobacillus* species, *G.vaginalis*, *Atopobium vaginae*, and *Mobiluncus* species significantly decreased the risk of cervical abnormalities versus NILM ‐*Lactobacillus* species and *G. vaginalis* significantly decreased the risk of cervical lesions versus NILM and ASC‐US ‐*Lactobacillus* species, *G. vaginalis*, *A. vaginae*, and *Mobiluncus* species significantly decreased the risk of HSIL/ASC‐H lesions versus NILM and ASC‐US and LSIL
[27]	Longitudinal (6+ visits)^b^	Canada	HPV acquisition	‐No significant association found between increasing RA of *Gardnerella swidinski* or *Lactobacillus crispatus* and the odds of incident hrHPV among HIV+ women
[28]	Longitudinal (2 visits)	Costa Rica	HPV clearance; cytology or biopsy results	‐Transition from a high to low *molBV* score had a significantly lower likelihood of hrHPV clearance compared to a sustained low score ‐Higher *molBV* score significantly increased the odds of hrHPV progression to CIN2+ lesions
[29]	Cross‐sectional	China	HPV prevalence; cytology or biopsy results	‐CST types found to significantly decrease the risk of hrHPV/lrHPV prevalence ‐No significant association found between CST types and CIN lesions
[30]	Nested‐case–control (2 visits)	Nigeria	HPV persistence	‐*Lactobacillus* dominance significantly decreased the odds of hrHPV persistence in HIV‐ women ‐*Lactobacillus* dominance increased the odds of hrHPV persistence in HIV+ women
[31]	Cross‐sectional	United States	HPV prevalence; cytology or biopsy results	‐No significant association between any CST type and the odds of hrHPV positivity or abnormal cytology ‐No significant association between the presence of *Lactobacillus gasseri* and hrHPV positivity or abnormal cytology following co‐variate adjustment
[32]	Longitudinal (3 visits)^c^	United States	Cytology or biopsy results	‐*Lactobacillus* depleted microbiome significantly associated with increased odds of non‐regression of CIN2 lesions after 12 and 24 months of follow‐up
[33]	Cross‐sectional	Korea	Cytology or biopsy results	‐Presence of *A. vaginae, Dialister invisus, Finegoldia magna, G. vaginalis, Prevotella buccalis*, and *Prevotella timonensis* significantly increased the odds of CIN2+ lesions or cervical cancer
[34]	Longitudinal (2 visits)	Costa Rica	Cytology or biopsy results	‐Increasing baseline RA of *Lactobacillus* significantly decreased the odds of progression to CIN2+ lesions ‐High microbial diversity at visit 2 significantly increased the odds of progression to CIN2+ lesions
[35]	Two nested‐case–control studies (both consisted of 2 visits)^b^	South Africa	HPV acquisition, clearance, prevalence, persistence; cytology or biopsy results	Among HIV+ women: ‐No significant association between the Simpson index, *L. crispatus* or *Lactobacillus jensenii* dominance, *Lactobacillus* RA, and abundance of BV anaerobes in baseline and/or samples at the end of follow up with the odds of hrHPV prevalence ‐A vaginal microbiome dominated by *L. crispatus* or *L. jensenii* in samples at the end of follow‐up significantly decreased the risk of an incident hrHPV infection ‐Higher microbial diversity, measured by the Simpson index in samples at the end of follow‐up, significantly increased the risk of hrHPV clearance and the risk of incident CIN2+ lesions and prevalence of CIN2+ lesions ‐A vaginal microbiome characterized by high diversity BV anaerobes at baseline significantly increased the risk of CIN2+ lesion clearance ‐Increasing BV‐anaerobes RA at baseline significantly increased the risk of CIN2+ lesions at one or both visits ‐Increasing *Lactobacillus* RA at the end of follow‐up significantly decreased the risk of CIN2+ lesions at one or both visits
[36]	Longitudinal (2 visits)	Brazil	HPV prevalence	‐Risk of HPV16 prevalence among women with a CST dominated by anaerobic bacteria was not significantly different from those with a CST dominated by *Lactobacillus iners* in pregnant women coinfected with HIV and HPV
[37]	Longitudinal (2–4 visits)	Korea	HPV clearance, prevalence, persistence	‐Increasing RA of *Eubacterium eligen, G. vaginalis*, and *Ureoplasma urealyticum* significantly increased the odds of hrHPV clearance ‐Increasing RA of *L. crispatus* significantly increased the odds of hrHPV negativity ‐Increasing RA of *Lactobacillus johnsonii* significantly increased the odds of hrHPV persistence
[38]	Cross‐sectional	China	Cytology or biopsy results	**‐**High and middle *Pseudomonas stutzeri* RA significantly decreased the odds of CIN2+ lesions ‐High *A.vaginae* RA significantly decreased the odds of CIN2+ lesions
[39]	Longitudinal (two visits)^c^	Italy	HPV persistence	‐CST IV‐BV significantly increased the odds of hrHPV persistence ‐CST IV‐AV significantly decreased the odds of hrHPV persistence
[40]	Cross‐sectional	Mexico	Cytology or biopsy results	‐No significant association found between high microbial diversity (Shannon diversity index and PD whole tree) and the odds of squamous intraepithelial lesions and cervical cancer
[41]	Cross‐sectional	Nigeria	HPV prevalence	‐No significant association between any CST type and the odds of hrHPV positivity
[42]	Cross‐sectional	United States	Cytology or biopsy results	‐A community type dominated by unclassified *Lactobacillus* and *L. iners* significantly associated with an increased risk of CIN2+ lesions among women with an hrHPV infection
[43]	Longitudinal (2–11 visits)	United States	HPV acquisition, prevalence	‐Transition to a CST dominated by *L. crispatus* significantly associated with a lower risk of incident HPV following adjustment for HIV study group ‐High RA of *L. crispatus* significantly associated with lower risk of any HPV and hrHPV detection among HIV+ /HIV‐ women
[44]	Cross‐sectional	Korea	Cytology or biopsy results	‐Dominance of *A. vaginae, G. vaginalis* and *L. iners* as well as the depletion of *L. crispatus* significantly associated with CIN risk ‐High RA of *A. vaginae* alone and with hrHPV negativity significantly associated with CIN risk
[45]	Longitudinal (25–33 visits)	United States	HPV acquisition, clearance	*‐*CST II significantly associated with more rapid clearance of HPV

Abbreviations: ASC‐H, atypical squamous cells – cannot exclude high‐grade squamous intraepithelial lesion; ASC‐US, atypical squamous cells of undetermined significance; BV, bacterial vaginosis; CIN, cervical intraepithelial neoplasia; CST, community state type; CVM, cervicovaginal microbiome; HIV, human immunodeficiency virus; HPV, human papillomavirus; hrHPV, high‐risk human papillomavirus; HSIL, high‐grade squamous intraepithelial lesion; ICC, invasive cervical cancer; lrHPV, low‐risk human papillomavirus; LSIL, low‐grade squamous intraepithelial lesion; *molBV* molecular bacterial vaginosis; NILM, negative for intraepithelial lesion or malignancy; PD, phylogenetic diversity; RA, relative abundance.

^a^ Cytology or biopsy results refer to cytological interpretations or biopsy‐confirmed CIN or cervical cancer as the outcome of interest.

^b^ Longitudinal data not collected for the entire study sample.

^c^ Corresponds to total visits as only baseline samples were used for microbial characterization.

#### 3.1.1 HPV Prevalence

Collectively, findings from the eight studies that assessed the relationship between the CVM and HPV prevalence generally suggest that *Lactobacilli* may be associated with a decreased risk of prevalent HPV infections and that high diversity may increase the risk [26, 29, 31, 35, 36, 37, 41, 43]. At the community level, CST type was identified as a significant predictor of HPV prevalence (adjusted odds ratio (aOR), 0.74; 95% CI, 0.59–0.93) [29]. Specifically, CSTs dominated by *Lactobacillus iners* (aOR, 0.67; 95% CI, 0.29–1.57 and aOR, 0.70; 95% CI 0.30–1.30), and *Lactobacillus crispatus* (aOR, 0.40; 95% CI, 0.10–1.70) reduced the odds of hrHPV, albeit insignificantly [31, 41]. Likewise, a community dominated by *L. crispatus* or *Lactobacillus jensenii* appeared to nonsignificantly decrease the risk of any hrHPV among women with human immunodeficiency virus (HIV) [35]. Three studies identified an increasing relative abundance (RA) of *Lactobacillus* species or *L. crispatus* to decrease the risk of hrHPV [35, 37, 43]. A reduction in the odds of several hrHPVs was associated with an increasing prevalence of *Lactobacillus* species (relative risk (RR) range, 0.50–0.69) [26], and the presence of *Lactobacillus gasseri* (aOR, 0.50; 95% CI 0.25–1.02) [31]. A high diversity CST appeared to nonsignificantly increase the odds of hrHPV (aOR, 1.53; 95% CI 0.62–3.76) [31]. However, in a different study among HIV/HPV co‐infected pregnant women, a CST dominated by anaerobic bacteria decreased the risk of HPV16 (RR, 0.75; 95% CI, 0.44–1.26), albiet insignificantly [36]. Other measures of bacterial diversity – the Simpson index and RA of BV anaerobes – suggested a nonsignificant increase in the risk of hrHPV among HIV‐positive women (relative risk ratio [RRR] range, 2.066–2.395) [35]. The presence of *Mobiluncus* species was significantly associated with an increased risk of HPV types targeted by the nine‐valent vaccine (RR, 1.85; 95% CI, 1.07–3.20) [26]. By contrast, diversity measured by prevalent BV bacteria and *Gardnerella vaginalis* appeared to significantly decrease the risk of multiple hrHPVs, HPV16 or 18, and 9‐valent HPV (RR range, 0.50–0.71) [26].

#### 3.1.2 HPV Acquisition

Temporal associations between the CVM and HPV acquisition were assessed by three longitudinal studies and one nested‐case–control study; *Lactobacillus* communities were often inversely associated with HPV incidence (effect estimate range, 0.125–0.910) [27, 35, 43, 45]. The aforementioned range excludes findings for a community dominated by *L. iners*, which appeared to imprecisely increase the risk of an incident HPV infection (adjusted transition rate ratio [aTRR], 1.79; 95% CI, 0.71–4.51) [45]. A decrease in the risk of hrHPV was found among HIV‐positive populations with a CVM dominated by *L. crispatus* or *L. jensenii* (RRR, 0.125; *p* = 0.019) [35]. An increasing RA of *L. crispatus* also decreased the odds of hrHPV (odds ratio (OR), 0.91; 0.84–1.01), although the estimate was not significant [27]. Similarly, a transition to a CST dominated by *L. crispatus* appeared to non‐significantly reduce the risk of an incident HPV infection (aTRR, 0.20; 95% CI, 0.03–1.14) among HIV‐positive and HIV‐negative subjects [43], whereas among the general population, there was a nonsignificant, inverse association between a CST dominated by *L. gasseri* and a transition from an HPV negative to a positive state (aTRR, 0.34; 95% CI, 0.06–1.85) [45].

#### 3.1.3 HPV Persistence

Longitudinal and nested‐case–control studies found associations between *Lactobacillus* species and microbial diversity and hrHPV persistence [30, 35, 37, 39]. The directionality of effect estimates for *Lactobacillus* species varied across studies and populations. A significant negative relationship was found between *Lactobacillus* species (≥ 70%) and hrHPV persistence (aOR, 0.35; 95% CI, 0.14–0.89) in HIV‐negative women, whereas in HIV‐positive women, a nonsignificant positive association with the odds of hrHPV persistence was evident (aOR; 1.25; 95% CI, 0.73–2.14) [30]. Conversely, in HIV‐positive women, a community dominated by *L. crispatus* or *L. jensenii* was associated, albeit not significantly, with a decreased risk of type‐specific hrHPV persistence (RRR, 0.315, *p* = 0.074) [35]. At the species level, among women with normal or atypical squamous cells of undetermined significance cytology, an increasing RA of *Lactobacillus johnsonii* appeared to significantly increase the odds of hrHPV persistence (aOR, 16.4; 95% CI, 1.77–152.2) [37]. Microbial diversity was generally indicative of an increased risk of persistence. CST IV‐BV (*Lactobacillus* species depleted with aerobic and anaerobic bacteria) was significantly associated with an increase in the odds of hrHPV persistence (aOR, 9.38; 95% CI, 1.85–47.52) [39]. Moreover, in women living with HIV, an increasing RA of BV anaerobes and the Simpson index were imprecisely associated with an increased risk of type‐specific hrHPV persistence [35]. Conversely, CST IV‐AV (*Lactobacillus* species depleted with strictly anaerobic bacteria) appeared to significantly decrease the odds of hrHPV persistence (aOR, 0.11; 95% CI, 0.01–0.93) [39].

#### 3.1.4 HPV Clearance

Four studies using longitudinal data (cohort or nested‐case–control studies) found significant associations between CVM components and HPV clearance [28, 35, 37, 45]. Two found lower diversity or *Lactobacillus* dominance to be significantly associated with HPV clearance [28, 45]. Usyk et al. assessed this association using a molecular BV score (*molBV*), where higher scores correspond to increased dysbiosis [28]. Individuals who transitioned from a high to low *molBV* score appeared to have a lower likelihood of hrHPV clearance relative to those with a consistently low score (adjusted hazard ratio; 0.55, 95% CI 0.30–0.97). Similarly, a CST dominated by *L. gasseri* was significantly associated with faster HPV clearance than one dominated by *L. crispatus* (aTRR, 4.43; 95% CI 1.11–17.7) [45]. Conversely, among HIV‐positive women, microbial diversity appeared to significantly increase the likelihood of hrHPV clearance by almost four times (RRR, 3.856; *p* = 0.034) [35]. One study identified individual species to be significantly associated with increased odds of hrHPV clearance: *Eubacterium eligen* (aOR, 11.5; 95% CI 1.31–101.4), *G. vaginalis* (aOR, 17.0; 95% CI 2.18–131.8), and *Ureaplasma urealyticum* (aOR, 7.42; 95% CI 1.30 – 42.5) [37].

#### 3.1.5 Associations with CIN and Cervical Cancer

Four of five cross‐sectional studies assessing associations between the CVM and biopsy‐confirmed lesions identified microbial components that significantly increased the risk of CIN or CIN2+ lesions [29, 33, 38, 42, 44]. CST type was not a significant predictor of CIN lesions (aOR, 1.09; 95% CI 0.85–1.41) [29], and the odds of CIN were approximately six times greater in women with a risky microbial pattern (defined by dominance of *Atopobium vaginae, L. iners, G. vaginalis* and depletion of *L. crispatus*) (aOR, 5.80; 95% CI 1.73–19.4) or a high RA of *A. vaginae* (aOR, 6.63; 95% CI 1.61–27.2) [44]. Dominance of *L. iners* and unclassified *Lactobacilli* appeared to significantly increase the odds of CIN2+ (aOR, 3.48; 95% CI 1.27–9.55) [42]. One study identified *A. vaginae, Dialister invisus, Finegoldia magna, G. vaginalis, Prevotella buccalis,* and *Prevotella timonensis* as bacterial species significantly associated with an increase in the odds of CIN2+ [33], whereas another suggested that *A. vaginae* and *Pseudomonas stutzeri* were associated with a significant decrease in the odds [38].

Findings varied across the four cross‐sectional studies that assessed the relation between the CVM and cytological interpretations [25, 26, 31, 40]. A diverse CST appeared to nonsignificantly increase the odds of abnormal cytology results (aOR, 1.63; 95% CI 0.66–4.03) [31], and high‐grade squamous intra‐epithelial lesions (SIL) or invasive cervical cancer (ICC) (aOR, 1.31; 95% CI 0.39–4.41) [25]. Similarly, microbial diversity (measured by an increasing Shannon index and phylogenetic diversity whole tree) appeared to nonsignificantly increase the odds of SIL or cervical cancer by over three times [40]. One study identified an imprecise inverse association between a CST dominated by *L. iners* and the risk of abnormal cytology (aOR, 0.67; 95% CI 0.28–1.59) [31], whereas another suggested it increased the odds of high‐grade SIL or ICC by approximately 13%, albeit insignificantly [25]. Only one study's findings – with assessment at the genus and species level – were statistically significant; *Lactobacillus* species, *G. vaginalis, A. vaginae* and *Mobiluncus* species were significantly associated with a decreased risk of cytological abnormalities [26].

Four longitudinal studies identified a significant relationship between high bacterial diversity or depletion of *Lactobacillus* species and progression or regression of CIN2+ lesions [28, 32, 34, 35]. High diversity – represented by an increasing *molBV* score, *Lactobacillus* depletion, the RA of BV‐anaerobes, the Shannon index, and the Simpson index – appeared to significantly increase the odds of CIN2+ prevalence, progression to CIN2+, non‐regression of CIN2+, or incident CIN2+ (effect estimate range, 1.17–7.352) [28, 32, 34, 35]. Accordingly, increasing baseline abundance of *Lactobacillus* was associated with a 59% decrease in the odds of CIN2+ lesion progression (aOR, 0.41; 95% CI 0.22–0.79) [34]. In contrast, high‐diversity BV anaerobes were significantly associated with a higher chance of CIN2+ clearance among women living with HIV [35].

#### 3.1.6 Systematic Reviews

Table 2 summarizes the main findings, which were consistent, from the four included systematic reviews published between 2018 and 2022 [46, 47, 48, 49]. A low abundance of *Lactobacillus* was related to HPV prevalence and low levels of *L. jensenii* and *Lactobacillus* have been found in the CVM of individuals with CIN and cervical cancer [49]. In the same review, high levels of *L. iners* and *Lactobacillus* species were also identified in CIN and cervical cancer. Another review that investigated the relationship between the vaginal microbiome and HPV infections found that high microbial diversity was related to CIN lesions and HPV acquisition, prevalence, and persistence but slower clearance of HPV [48]. Sims et al. and Gardella et al. evaluated the role of the CVM in CIN and cervical cancer [46, 47]. Generally, both reviews suggested that dysbiosis and a reduction in *Lactobacillus* species promote HPV‐associated cervical carcinogenesis via their association with either HPV acquisition, prevalence, persistence, clearance, and cytology or biopsy‐confirmed CIN or cervical cancer.

**Table 2:** Systematic reviews on the relationship between the CVM and HPV prevalence, acquisition, persistence, clearance, and/or cytology interpretations or biopsy‐confirmed CIN and cervical cancer.

References	Focus (sample size [*n*] of studies)	Microbiome characterization^a^	Outcome(s) assessed^b^	Main findings^c^	Limitations acknowledged
[46]	Interplay between HPV‐associated CIN, the vaginal microbiome and the immune system (*n* = 6)	Wet‐mount microscopy, QiAMp Mini DNA kit, 16S rRNA, PowerSoil DNA Isolation Kit	HPV prevalence, persistence; cytology or biopsy results	‐The following were found to be linked to an HPV infection: (1) high diversity, (2) loss of *Lactobacillus*, (3) CST IV, (4) *Shuttleworthia*, (5) *Gemella*, (6) *Olsenella*, (7) common bacterial vaginosis bacteria (*Gardnerella, Prevotella, Atopobium, Megasphaera, Parvimonas, Anaerococcus, Peptostreptococcus, Sneathia*), and (8) aerobic vaginitis and other dysbiosis microbes	‐None reported
				‐Relationship between dysbiosis, high diversity, and/or *Lactobacillus* depletion and HPV persistence	
				‐In HSIL and/or cervical cancer, (1) *Lactobacillus* depletion, (2) increase in anaerobes, and (3) bacterial vaginosis are evident ‐*Shuttleworthia, Gemella, Olsenella, Gardnerella, Prevotella, Atopobium, Megasphaera, Parvimonas, Anaerococcus, Peptostreptococcus, Sneathia* were related to high‐grade and low‐grade lesions and invasive cervical cancer ‐Vaginal dysbiosis may occur in low‐grade and high‐grade disease and *Sneathia sanguinegens* may be more frequent in high‐ than low‐grade lesions ‐*Lactobacillus iners* may be associated with risk of cervical dysplasia ‐Increased expression of *Lactobacillus jensenii* and *Lactobacillus coleohominis* is evident in LSIL patients ‐Risk of CST IV (high diversity) may increase with increasing lesion severity (LSIL, HSIL, and invasive cancer) and risk of lesion severity was lower with CST I (*Lactobacillus crispatus* dominance) ‐Transition from CST IV (high diversity) to CST II (*Lactobacillus gasseri*) observed in CIN ‐Patients with LSIL mainly have a microbial pattern of CST I (*L. crispatu*s) ‐Aerobic vaginitis associated with cervical dysplastic lesions ‐*Sneathia* species may serve as a biomarker for CIN progression and *Delftia* species for squamous intraepithelial lesions ‐Inconclusive findings in the literature on the association between BV and cervical cancer development	
[47]	Cervicovaginal and gut microbiome in CIN and Cervical Cancer(*n* = 8)^d^	Gram staining, 16S rRNA, WMS	HPV prevalence, acquisition, persistence; cytology or biopsy results	‐*L. crispatus* dominance associated with lower HPV prevalence ‐*Lactobacillus* species produce lactic acid and a depletion in their abundance could result in an inability to fight off viruses (i.e., HPV)	‐None reported
				‐Dysbiosis and high diversity may increase the risk of hrHPV	
				‐Together, high diversity, dysbiosis, and inflammation can facilitate HPV persistence ‐High diversity linked to HPV persistence	
				‐Reduction in *Lactobacillus* species may result in carcinogenic changes ‐CST of women with CIN may be: (1) *Lactobacillus* depleted, (2) dominated by anaerobic bacteria, and (3) dominated by *L. iners* ‐High bacterial diversity associated with CIN lesion severity ‐*Sneathia* species linked to CIN lesions ‐Microbial composition is similar amongst BV and cervical cancer patients ‐*Fusobacterium* found in CIN and cervical cancer, and more common in cervical cancer	
[48]	Relationship between the vaginal microbiome and HPV(*n* = 19)^e^	Microarray technology, microscopic evaluation, PCR‐DEJA assay, microbiological cultures, vaginal pH, 16S rRNA, 16S rDNA	HPV prevalence, acquisition, persistence, clearance; cytology or biopsy results	‐HPV‐positive women may have (1) a high diversity microbiome, (2) depletion of *Lactobacillus* species, (3) lower abundance of *L. iners and L. crispatus*, (4) increase in anaerobic bacteria (i.e., *Prevotella* and *Leptotrichia*) ‐*Gardnerella vaginalis* and *L. gasseri* are common in HPV‐positive patients ‐Studies found a relationship between *L. crispatus* and a lower prevalence of hrHPV	‐Number of existing studies and subsequently patients included in the synthesis is limited ‐Abstracts, reviews, conference papers, editorials, animal studies and commentaries were not considered
				‐CST IV‐A (high diversity) may be related to a transition from an HPV‐negative to an HPV‐positive state compared to CST I (*L. crispatus* dominant)	
				‐CST III (*L. iners* dominant) and CST IV‐B (high diversity) may result in faster and slower clearance of HPV, respectively	
				‐CST IV‐B (high diversity) may increase the risk of HPV persistence	
				‐CST III (*L. iners* dominant) has been related to severe CIN lesions ‐CIN lesions linked to (1) high diversity with *Sneathia* species, and (2) increased abundance of *Lactobacillus and Lactobacillus reuteri* (specifically in CIN2) ‐*Fusobacterium* species frequently detected in women with cervical cancer	
[49]	Role of *Lactobacillus* species in cervical cancer(*n* = 29)	16S rRNA, 16S rDNA, PCR, microscopic evaluation, bacterial isolation, and purification^f^	HPV prevalence; cytology or biopsy results	‐Lower abundance of *Lactobacillus* species in HPV‐positive women	‐ The mechanistic role of *Lactobacillus* in cervical cancer is ill‐defined ‐Paucity of existing RCTs and cohort studies on the topic ‐Unpublished research and ongoing studies were not considered
				‐High RA of *Lactobacillus* sp. and *L. iners* in precancerous lesions and cervical cancer ‐Low RA of *L. jensenii* and *L. crispatus* in precancerous lesions and cervical cancer	

Abbreviations: CIN, cervical intraepithelial neoplasia; CST, community state type; HPV, human papillomavirus; hrHPV, high‐risk human papillomavirus; HSIL, high‐grade squamous intraepithelial lesions; LSIL, low‐grade squamous intraepithelial lesions; PCR, polymerase chain reaction; RA, relative abundance; RCT, randomized controlled trial; sp, singular species; WMS, whole metagenome sequencing; 16S rRNA, 16S ribosomal RNA; 16S rDNA, 16S ribosomal DNA.

^a^ Refers to the microbial characterization techniques utilized by the studies included in the systematic review.

^b^ Cytology or biopsy results refer to cytological interpretations or biopsy‐confirmed CIN or cervical cancer as the outcome of interest.

^c^ Main findings are subdivided according to the outcomes assessed.

^d^ Included studies do not include those that assessed the relationship between the gut microbiome and cervical cancer.

^e^ Discrepancy whereby authors listed 19 studies in Table 1 but state that only 16 met their inclusion criterion.

^f^ This systematic review included findings from both clinical and experimental studies; the tabulated microbial characterization techniques refer to those reported for the clinical studies.

#### 3.1.7 Meta‐Analyses

Table 3 details the main components from all three meta‐analyses published in 2019 [18, 19, 20]. One provided separate effect estimates from studies that used microscopic and molecular characterization techniques; based on findings from the two molecular studies (with no heterogeneity; *I* ^2^ = 0.0%), low *Lactobacilli* anaerobes appeared to significantly increase the odds of a persistent HPV infection (RR, 2.00; 95% CI, 1.05–3.81) [18]. Two meta‐analyses assessed the relationship between the CVM and HPV prevalence [19, 20]. Low *Lactobacillus* species appeared to significantly increase the odds of an HPV infection based on 20 studies (with substantial heterogeneity; *I* ^2^ = 66.1%) (OR, 1.53; 95% CI 1.23–1.82) [19]. Similarly, based on nine studies (with low heterogeneity; *I* ^2^ = 6.0%), a CST dominated by *Lactobacillus* species significantly decreased the odds of hrHPV (OR, 0.64; 95% CI 0.48–0.87) [20]. Moreover, based on 8 studies (with low heterogeneity; *I* ^2^ = 10.0%), the same meta‐analysis found a *L. crispatus*‐dominant CST to be significantly associated with lower odds of hrHPV prevalence [20]. With respect to CIN lesions, a *Lactobacillus* dominant CST (*n* = 6 studies, with no heterogeneity; *I* ^2^ = 0.0%) and a CST dominated by *L. crispatus* (*n* = 5 studies, with no heterogeneity; *I* ^2^ = 0.0%) were significantly associated with a decreased odds of CIN lesions by approximately 47% and 50%, respectively [20]. In the same meta‐analysis, a *Lactobacillus* dominant CST significantly decreased the risk of cervical cancer based on findings from three studies (with no heterogeneity; *I* ^2^ = 0.0%) [20]. None of the identified meta‐analyses assessed the association between the CVM and HPV clearance. Brusselaers et al. showed that non‐*L. crispatus* dominance may increase the risk of HPV acquisition by almost twofold based on two molecular studies (with moderate heterogeneity; *I* ^2^ = 55.7%); however, the estimate was not statistically significant [18].

**Table 3:** Meta‐analyses on the association between the CVM and HPV prevalence, acquisition, persistence, clearance, and/or cytology interpretations or biopsy‐confirmed CIN and cervical cancer.

References (sample size [*n*] of studies)	Microbiome characterization^a^	Inclusion criteria *Exclusion criteria*	Exposure	Outcome	Overall effect estimate (95% CI) *I* ^2^	Limitations acknowledged
[18]^b^ (*n* = 15), but tabulated risk estimates were based on two studies using molecular techniques	Microscopy (Nugent Scoring, wet mount microscopy, Pap‐stained smears) and Molecular (16S rRNA and cpn60 gene sequencing)	Original research, longitudinal or nested‐case–control studies; Compare women with/without vaginal dysbiosis and assessed risk of HPV incidence, persistence and/or SIL lesions; Minimum of 2 measurement points; Microbial assessment via microscopic or molecular methods *Microbial assessment via vaginal pH measurement*	Non‐*Lactobacillus crispatus* dominant	Incident HPV	RR, 1.85 (0.47–7.32) 55.7%	‐Confounding ‐Differences in exposures/outcomes, study populations, and HPV types ‐Misclassification bias ‐Potential for reverse causation
			Non‐*L. crispatus* dominant	Persistent HPV	RR, 1.33 (0.63–2.81) 23.8%	
			*Lactobacillus gasseri* dominant	Persistent HPV	RR, 0.63 (0.10–3.86) 81.0%	
			*Lactobacillus iners* dominant	Persistent HPV	RR, 1.06 (0.42–2.63) 0.0%	
			Low *Lactobacilli*, mixed aerobe and anaerobe	Persistent HPV	RR, 1.00 (0.23–4.30) 80.1%	
			Low *Lactobacilli* anaerobe	Persistent HPV	RR, 2.00 (1.05–3.81) 0.0%	
[19] (*n* = 39) but risk estimate tabulated was based on 20 studies	16S rRNA gene amplicon sequencing, Nugent Score, Amsel's criteria, or presence of clue cells	Association between vaginal microbiota and HPV, *C. trachomatis, M. genitalium* and/or *N. gonorroeae*; Microbial characterization via 16S rRNA gene amplicon sequencing, Nugent score, Amsel's criteria or presence of clue cells; Human study population; Cohort, cross‐sectional or interventional study; Detect STIs with PCR *HIV positive (study population or* > *10% of participants); Pregnant; Literature review, letters and/or editorials; Sample size* < *30*	Low *Lactobacillus* vs. high *Lactobacillus* vaginal microbiome	HPV Infection	OR, 1.53 (1.23–1.82) 66.1%	‐Methods for STI diagnoses have different sensitivity and specificity ‐Different methods for microbial characterization ‐Pooling methods could mask the effect of individual species on STIs ‐No overall measure of association ‐Publication bias
[20] (*n* = 11) but risk estimate tabulated differed by exposure/outcome considered	PCR amplification of 16S rRNA gene sequencing, Microarray with probes targeting 16S, and 18S rRNA genes	Association between cervicovaginal *Lactobacilli* and hrHPV/CIN/cervical cancer *Lactobacillus examination with microscopic techniques; Lactobacillus had to be assessed quantitatively in CST* ^c^; *Studies in human cell lines or animal models; Abstracts; Non‐English articles; Duplicate publications; Studies without numerical value of raw data*	*Lactobacillus* dominant *CST*	hrHPV	OR, 0.64 (0.48–0.87), based on 9 studies 6.0%	**‐**Cross‐sectional analysis ‐Small number of studies and sample size ‐Variation in 16S hypervariable regions ‐Analysis restricted to CST types
			*L. iners* dominant CST III	hrHPV	OR, 0.96 (0.69–1.34), based on 8 studies 0.0%	
			*L. crispatus* dominant CST I	hrHPV	OR, 0.49 (0.31–0.79), based on 8 studies 10.0%	
			*Lactobacillus* dominant CST	CIN	OR, 0.53 (0.34–0.83), based on 6 studies 0.0%	
			*L. iners* dominant CST III	CIN	OR, 0.99 (0.60–1.64), based on 5 studies 0.0%	
			*L. crispatus* dominant CST I	CIN	OR, 0.50 (0.29–0.88), based on 5 studies 0.0%	
			Cervicovaginal *Lactobacillus* pre‐dominant CST	Cervical Cancer	OR, 0.12 (0.04–0.36), based on 3 studies 0.0%	
			*L. iners* dominant CST III	Cervical Cancer	OR, 0.13 (0.02–1.13), based on 2 studies 0.0%	
			*L. crispatus* dominant CST I	Cervical Cancer	OR, 0.17 (0.03‐1.05), based on 2 studies 0.0%	

Abbreviations: CIN, cervical intraepithelial neoplasia; CST, community state type; HIV, human immunodeficiency virus; HPV, human papillomavirus; hrHPV, high‐risk human papillomavirus; OR, odds ratio, PCR, polymerase chain reaction; RR, relative risk; SIL, squamous intraepithelial lesion; STI, sexually transmitted infection; 16S rRNA, 16S ribosomal RNA; 18S rRNA, 18S ribosomal RNA.

^a^ Refers to the microbial characterization techniques utilized by the studies included in the meta‐analysis.

^b^ This meta‐analysis included findings from studies that characterized the microbiome using molecular and microscopic techniques. We only tabulated the effect estimates for molecular studies.

^c^ This exclusion criterion was only for studies included in the quantitative synthesis.

### 3.2 Diagnostic Ability of the CVM in HPV‐Associated Cervical Carcinogenesis

Three cross‐sectional studies, published in 2020 or 2021, assessed the diagnostic performance of CVM components to detect HPV (*n* = 2) [50, 51] or high‐grade cervical lesions (*n* = 1) [52], the details of which are presented in Table 4. All detected the outcomes of interest with high accuracy, as demonstrated by their respective AUCs. Among 546 women of reproductive age in Brazil, 30 bacterial taxa were strongly correlated with hrHPV (AUC = 0.802; 95% CI, 0.752–0.853) [50]. Similarly, among 52 women attending a colposcopy clinic following screening for cervical cancer, 17 genera (AUC = 0.819; 95% CI, 0.684–0.954) and seven species (AUC = 0.918; 95% CI, 0.839–0.997) had good clinical performance to detect HPV16 positivity [51]. The abundance of 33 bacterial species was extremely able to discriminate between CIN2+ and CIN1‐ lesions among 66 women in Korea (AUC = 0.952; 95% CI, 0.820–1.000), however, the estimate was not statistically significant [52].

**Table 4:** Diagnostic performance of the CVM to detect HPV and high‐grade lesions in studies reporting a receiver operating characteristic curve analysis.

References	Study Design Population (sample size [*n*] of participants; *breakdown by outcome*) Age	Microbiome characterization Hypervariable region Sequencing	HPV genotyping method Types Detected	Analysis approach to select microbial components *Outcome*	Number of microbial components included *Enumeration of microbial components*	AUC (95% CI)
[50]	Cross‐sectional Reproductive‐aged women in Brazil (*n* = 546; *86 hrHPV*+, *460 hrHPV−)* Ages, 18–51 years	16S rRNA V3–V4 Illumina MiSeq	Linear Array HPV genotyping hrHPVs; 16, 18, 26, 31, 33, 35, 45, 51, 52, 53, 56, 58, 59, 66, 68, 69, 70, 73, and 82	Multivariable logistic regression with a stepwise forward selection algorithm (*p* < 0.15 for variable retention) *hrHPV Infection*	30 out of 116 bacterial taxa *Shuttleworthia satelles, Sutterella stercoricanis, Peptoniphilus, Eubacterium saphenum, Lactobacillus salivarius, Sutterella morbirenis, Pediococcus acidilactici, Aerococcus viridans*, BVAB3, *Prevotella* genogroup 3, *Streptococcus intermedius, Corynebacterium accolens, Dialister* sp type 2, *Megasphaera* sp type 2, *Dialister propionicifaciens*, *Eubacterium siraeum, Bacteroides uniformis, Prevotella* genogroup 2, *Leptotrichia amnionii, Acinetobacter calcoaceticus, Arcanobacterium hippocoleae, Roseburia intestinalis, Porphyromonas endodontalis, Enterococcus faecalis, Varibaculum cambriense, Raoultella planticola, Staphylococcus lugdunensis, Streptococcus anginosus, Mycoplasma genitalium, Streptococcus mutans*	0.802 (0.752–0.853)
[52]	Cross‐sectional Healthy women and those with CIN in Korea (*n* = 66; *42 CIN2*+, *24 CIN1−*)^a^ Age, mean 45.1	16S rRNA V3 Ion Torrent PGM for 1250 flows with Ion PGM Hi Q Sequencing Kit	Anyplex II HPV 28 assay kit 28 HPVs; 18 hrHPVs and 8 lrHPVs hrHPVs: 16, 18, 26, 31, 33, 35, 39, 45, 51, 52, 56, 58, 59, 66, 68, 69, 73, and 82 lrHPVs: 6, 11, 40, 42, 44, 53, 54, and 70	Random forest model using grid search on a five‐run 10‐fold cross‐validation to select top 40 optimal bacterial species which were gradually added in the final model *CIN2+ versus CIN1−*	Abundance of 33 bacterial species *Lactobacillus iners, Gardnerella vaginalis, Atopobium vaginae, Lactobacillus gasseri, Ureaplasma parvum, Streptococcus agalactiae, Lactobacillus amylovorus, Streptocccus anginosus, Lactobacillus casei, Lactobacillus helveticus, Lactobacillus acetolerans, Lactobacillus acidophilus, Prevotella disiens, Aerococcus christensenii, Lactobacillus vaginalis, Bifidobacterium dentium, Atopobium minutum, Lactobacillus johnsonii, Finegoldia magna, Lactobacillus pontis*, Unclassified *Dialister, Corynebacterium tuberculostearicum, Sneathia sanguinegens, Lactobacillus jensenii, Streptococcus salivarius, Enteroccocus faecalis, Prevotella bivia, Lactobacillus delbrueckii subsp Bulgaricus*, Unclassified *Megasphaera*, *Prevotella timonensis*, Unclassified *Prevotella*, *Streptococcus canis*, *Clostridium* BVAB2	0.952 (0.820–1.000)
[51]	Cross‐sectional Women attending a colposcopy clinic following screening for cervical cancer (*n* = 52; *27 HPV16* + , *25 HPV−*) Ages, 25–42 years	Shotgun metagenomic sequencing NA Hiseq X‐ten platform	Hybribio Rapid GenoArray test kit 15 hrHPVs, 6 lrHPVs hrHPVs: 16, 18, 31, 33, 35, 39, 45, 51, 52, 56, 58, 59, 68, 66, and 53 lrHPVs: 6, 11, 42, 43, 44, and CP8304	Random forest ensemble learning based on the mean decrease accuracy *HPV16+ versus HPV−*	17 genera NR^b^	0.819 (0.684–0.954)
					7 species *NR* b	0.918 (0.839–0.997)

Abbreviations: AUC, area under the curve; CIN, cervical intraepithelial neoplasia; HPV, human papillomavirus; hrHPV, high‐risk HPV; lrHPV, low‐risk HPV; NA, not applicable; NR, not reported; 16S rRNA, 16S ribosomal RNA.

^a^ CIN2+ group consisted of CIN2 lesions to cervical cancer whereas CIN1− consisted of healthy controls to CIN1 lesions.

^b^ Putative genera and species were listed in the article but those used in ROC analysis were not specified.

### 3.3 16S rRNA Gene Sequencing and WMS

Most research articles in the narrative synthesis utilized 16S rRNA gene sequencing (*n* = 20) for CVM characterization. Table 5 compares the identification ability, processes, advantages, and disadvantages of 16S rRNA gene sequencing with those of WMS. For CVM characterization, both techniques begin with the collection of a cervical or vaginal sample and extraction of genomic DNA. 16S rRNA gene sequencing relies on amplifying different hypervariable region(s) (i.e., amplicon) of the 16S rRNA bacterial gene (a subunit of a ribosome found in all bacteria and archaea) using PCR, whereas WMS relies on all genomic DNA. High‐throughput sequencing analyzed with a bioinformatics pipeline allows for assessing a sample's bacterial (16S rRNA) and microbial (WMS) diversity. The major difference between the two techniques is that WMS is more comprehensive, as it has the ability to allow isolate‐level resolution and detect the complete microbiome consisting of several micro‐organisms including archaea, bacteria, eukaryotes, viruses, fungi, and plasmids. By contrast, 16S rRNA gene sequencing can only detect archaea and bacteria that contain the primer set specific sequences in their genome. Moreover, the choice of primer set is an important determinant of species to be detected in the CVM [63, 64]. Notwithstanding, both WMS and 16S rRNA, could in principle identify the bacteriome [57]. Although more comprehensive, WMS is expensive and generates large amounts of data. Despite limited taxonomic resolution, 16S rRNA gene sequencing is cost‐effective and well‐established as a starting point for microbial identification.

**Table 5:** 16S rRNA gene sequencing and WMS for CVM characterization.

Features	16S rRNA Gene Sequencing	WMS
Technique and process [53, 54, 55, 56]	Collection of cervical and/or vaginal sample	
	Extraction of genomic DNA from the cervical and/or vaginal sample	
	PCR amplification of hypervariable region(s) of the 16S rRNA bacterial gene	DNA shearing of all genomic DNA
	—	Preparation of WMS library depending on the target organism(s) and DNA fragmenting
	Amplicon sequencing using a sequencing platform (i.e., Pyrosequencing, Illumina MiSeq, Illumina nextSeq, PacBlo Seqel II/IIe, Nanopore)	Sequencing all genomic DNA using a sequencing platform (i.e., Illumina novaSeq, HiSeq**)**
	Taxonomic assignment using curated 16S rRNA databases	Taxonomic assignment using nonredundant databases or marker databases (metaphlan)
	Assessment of RA and diversity of taxon between samples	Bioinformatics analysis to assess the microbial makeup of a sample
Ability to identify specific microbes [24]	Bacteriome (i.e., bacteria present in a sample at the genus and/or species level)	Complete microbiome (i.e., archaea, bacteria, eukaryotes, viruses, fungi, and plasmids)
CVM Identification [57]	Bacteriome	
Advantages *Disadvantages* [58, 59, 60, 61, 62]	Cost‐effective, time‐efficient, well‐established databases *Generally limited taxonomic resolution of standard bioinformatics pipelines*	Comprehensive, high taxonomic resolution, identifies variants, and mutations *Large quantity of noisy data, expensive, timely, databases are relatively novel*

Abbreviations: PCR, polymerase chain reaction; RA, relative abundance; WMS, whole metagenome sequencing; 16S rRNA, 16S ribosomal RNA.

## 4 Discussion

Findings from our review may aid in understanding associations between the CVM and HPV prevalence, acquisition, persistence, clearance, and pre‐cancerous lesions and cervical cancer. To the best of our knowledge, this is the first review on the topic that only included studies that used metagenomic methods without restricting to specific microbes and summarized the diagnostic performance of CVM communities. Our findings from the assessment of individual studies were aligned with previous systematic reviews assessing similar relationships [46, 47, 48, 49]. Although associations were not consistent across studies, we identified a general trend of *Lactobacillus* communities decreasing the risk of adverse outcomes in HPV‐associated cervical carcinogenesis, whereas high diversity appeared to increase the risk. Findings for specific microbial components were irreconcilable; studies identified unique species and demonstrated different associations, based on both directionality and statistical significance, with the main outcomes. Additionally, distinct combinations of microbial components were strongly correlated with and able to detect HPV16 [51], hrHPV [50], and/or CIN lesions [52].

The findings suggest that several species may be implicated in HPV‐associated cervical carcinogenesis. At the level of individual species, *L. crispatus* [37, 43], *E. eligen* [37], *G. vaginalis* [26, 37], *U. urealyticum* [37], *P. stutzeri* [38], and *A. vaginae* [26, 38] significantly increased the risk of the outcomes of interest. On the other hand, *A. vaginae* [33], *D. invisus* [33], *F. magna* [33], *G. vaginalis* [33], *P. buccalis* [33], *P. timonensis* [33], and *L. johnsonii* [37] significantly decreased the risk. The role of additional species remains inconclusive as estimates were not statistically significant. Conflicting findings at the species level suggest that a microbial community rather than a single species may contribute to cervical carcinogenesis. Alternatively, this could highlight a need for a comprehensive assessment of bacterial species using high‐resolution characterization methods. Nevertheless, a communal role is supported by the observation that a CVM dominated by *Lactobacillus* species often significantly decreases the risk of adverse outcomes in HPV‐associated cervical carcinogenesis [20, 26, 30, 34, 35], whereas high diversity (or a loss of *Lactobacillus* dominance) often significantly increases the risk [18, 19, 28, 32, 34, 35, 39, 44]. Furthermore, *Lactobacillus* species sustain a low vaginal pH due to the production of lactic acid [65], and some *Lactobacilli* produce lactocin (a bacteriocin) [66]. Both of these substances may help to maintain a healthy vaginal environment, further contributing to the plausibility of a communal role in cervical carcinogenesis.

The CVM of reproductive‐aged women is thought to be dominated by *Lactobacillus* species and generally represents a healthy state, whereas diversity indicates a diseased one [9, 10]. However, CVM composition has been shown to vary by ethnicity. Among women in Amsterdam, a high diversity CVM consisting of *G. vaginalis* was more common in those of sub‐Saharan African ancestry than Dutch [67]. Hence, a unique genetic or environmental factor pertaining to country of origin may impact the predisposition of the presence and favored harboring of certain CVM microbes. The identified studies looking at the association between the CVM and HPV‐associated cervical carcinogenesis were conducted in various countries and, in some cases, specific ethnicities; it is plausible to assume that ethnicity varied greatly between studies. The inconsistency in effect estimates and the lack of inter‐study reproducibility may result from incomparable study populations concerning the country of origin and differing microbial compositions. Accordingly, Tossas et al. 2023, have demonstrated that CVM composition modifies the association between race and CIN3; black women with a suboptimal CVM had an approximately eightfold increase in the risk of CIN3 compared to white women [68].

Several screening modalities have been implemented for cervical cancer. Historically, cervical cytology was the paradigm for screening. Despite its high test specificity for the detection of pre‐cancerous lesions (96%–98%), its sensitivity is poor (51%–53%) [69, 70, 71], highlighting a need for repeat testing to minimize false negatives. Comparatively, HPV DNA testing has been shown to have a higher sensitivity (94.6%) and slightly lower specificity (94.1%) for detecting high‐grade lesions [72]. By combining cytology and HPV primary testing, the specificity and sensitivity are maximized. We identified three studies suggesting that the presence and/or RA of bacterial taxa can robustly detect HPV [50, 51] or CIN lesions [52]. If the validity measurements for the CVM detecting pre‐cancerous lesions outweigh those of HPV DNA testing or cytology alone, microbial testing could be a feasible and cost‐effective option for cervical cancer screening. Nevertheless, further research is required to identify specific CVM components, including reproducibility in longitudinal data to establish causality.

Most observational studies we identified utilized 16S rRNA gene sequencing to identify microbial communities and explore associations with the outcomes of interest. Variation exists within 16S rRNA gene sequencing, and the identified observational studies used different sequencing platforms and bioinformatics annotation pipelines and amplified at various hypervariable regions. It is reasonable to hypothesize that both the diversity in the identified bacterial communities and variation in CVM exposure assignment across investigations could be attributable to these methodological differences. Few studies were able to examine the same relationships between the CVM and outcomes of interest due to varying exposures. Thus, to enable scientific replicability and confirm associations across studies, it may be important to establish a harmonious CVM characterization technique to identify similar bacterial communities. Standardization of the methodological procedures within 16S rRNA gene sequencing may be a feasible option. Although this method is not the most comprehensive for microbial identification as it targets bacterial communities [24], we identified literature showing that it detects similar CVM microbial species as WMS [57]. The latter technique can identify the complete microbiome [24]. This detection comparability suggests that bacteria may be the main constituent of the CVM. As such, the cost‐effectiveness and efficiency of 16S rRNA gene sequencing render it a potential option for CVM characterization.

We comprehensively summarized the current literature on the CVM and HPV‐associated cervical carcinogenesis. Another inherent strength was the restriction to studies that characterized the CVM using metagenomic methods, thus enabling comparison of findings across studies. However, several considerations need to be taken into account. First, among the included observational studies, there was a large variation in sample size, HPV genotyping methods, microbial characterization methods, and covariate adjustment. Several of the effect estimates had large CIs, and thus, the reported associations should be interpreted with caution. Such imprecision is a common limitation in microbiome studies which are often based on small sample sizes due to the high costs of microbial characterization and the large number of microbes detected in comparison to the number of observations. Exposure misclassification may have arisen in studies using 16S rRNA gene sequencing due to limited taxonomic resolution and errors across 16S reference databases. As a result, some participants may have been mistakenly categorized with respect to their microbiome profile, which may bias the directionality of effect estimates. Second, in terms of technical aspects related to characterization of the CVM, the choice of primer sets for amplification with 16S rRNA gene sequencing may have resulted in the identification of different bacterial species leading to incomparable study populations with respect to the microbiome. Third, our findings may be limited by the inclusion of several cross‐sectional studies in which temporal changes in CVM composition were not observed and thus causation cannot be established. Moreover, two longitudinal studies only characterized the CVM in baseline samples; changes in microbial variability were not examined over time concerning hrHPV persistence and CIN lesion regression [32, 39]. Consequently, there is a need for more longitudinal studies to infer causality or explore whether the CVM could mediate the relationship between HPV infections and cervical cancer. Fourth, we did not explore the role of archaea, fungi, plasmids, or viruses in HPV‐associated cervical carcinogenesis. However, the majority of identified studies utilized 16S rRNA gene sequencing for CVM characterization, a technique designed to specifically target the bacteriome. Finally, we may have missed identifying important literature by excluding abstracts and articles published in languages other than English. Nevertheless, our search was developed with the assistance of a librarian, and we comprehensively searched three databases.

To conclude, although there is suggestive evidence for the involvement of the CVM in HPV‐associated cervical carcinogenesis, the observed conflicting findings and imprecise estimates reported across studies that characterized the CVM using metagenomic techniques warrant consideration. Cost‐effective methodological techniques for CVM characterization and sampling approaches to control for intra‐individual CVM variation over time are needed to aid in establishing the role of the CVM in HPV‐associated cervical carcinogenesis.

## Author Contributions

Margaret Logel, Mariam El‐Zein, and Eduardo L. Franco formulated the research question. Margaret Logel created the searches and performed primary screening for the epidemiological associations. Margaret Logel and Parker Tope performed secondary screening and extracted data from relevant records. Margaret Logel performed all screening and extraction for the diagnostic performance. Margaret Logel drafted the manuscript under the supervision of Mariam El‐Zein, Emmanuel Gonzalez, and Eduardo L. Franco. Parker Tope, Mariam El‐Zein, Emmanuel Gonzalez, and Eduardo L. Franco reviewed and amended the manuscript. All authors read and approved the final version of the manuscript.

## Conflicts of Interest

Eduardo L. Franco reports grants and/or consulting fees from Merck and the Canadian Institutes of Health Research (CIHR), outside of the submitted work. Eduardo L. Franco and Mariam El‐Zein hold a patent related to the discovery of “DNA methylation markers for early detection of cervical cancer”, registered at the Office of Innovation and Partnerships, McGill University, Montreal, Quebec, Canada (October 2018). Eduardo L. Franco is an Advisory Board Member of Cancer Research Organizations (Camargo Cancer Center, Charbonneau Cancer Institute, Universidade do Minho). Eduardo L. Franco receives an honorarium as an editor for various journals (Oxford University Press, Elsevier and Elifesciences Ltd.). Mariam El‐Zein receives an honorarium from Elsevier as an Associate Editor for Preventive Medicine. Margaret Logel and Parker Tope receive an honorarium from Elsevier as an Assistant Editor for Preventive Medicine and Preventive Medicine Reports. Margaret Logel was awarded a master's fellowship by Fonds de Recherche du Québec – Santé. Emmanuel Gonzalez has nothing to disclose.

## Supporting information

Supporting information.

## Data Availability

Data sharing is not applicable to this article as no new data were created or analyzed in this study. Data availability and sharing are not applicable to this manuscript as we used secondary data from the published literature; no datasets were generated or analyzed.
